# Household transmission of seasonal coronavirus infections: Results from the Flu Watch cohort study

**DOI:** 10.12688/wellcomeopenres.16055.1

**Published:** 2020-06-19

**Authors:** Sarah Beale, Dan Lewer, Robert W. Aldridge, Anne M. Johnson, Maria Zambon, Andrew Hayward, Ellen Fragaszy

**Affiliations:** 1 UCL Public Health Data Science Research Group, UCL Institute of Health Informatics, University College London, London, NW1 2DA, UK; 2 UCL Research Department of Epidemiology & Public Health, University College London, London, WC1E 7HB, UK; 3 UCL Institute for Global Health, University College London, London, WC1E 7HB, UK; 4 NIHR Health Protection Research Unit in Respiratory Infections, Imperial College London, London, W2 1PG, UK; 5 Public Health England, London, EC4Y 8AE, UK; 6 Department of Infectious Disease Epidemiology, London School of Hygiene and Tropical Medicine, London, WC1E 7HT, UK

**Keywords:** coronavirus, HCoV-NL63, HCoV-OC43, HCoV-229E, SARS-CoV-2, epidemiology

## Abstract

**Background:** In the context of the current coronavirus disease 2019 (COVID-19) pandemic, understanding household transmission of seasonal coronaviruses may inform pandemic control. We aimed to investigate what proportion of seasonal coronavirus transmission occurred within households, measure the risk of transmission in households, and describe the impact of household-related factors of risk of transmission.

**Methods: **Using data from three winter seasons of the UK Flu Watch cohort study, we measured the proportion of symptomatic infections acquired outside and within the home, the household transmission risk and the household secondary attack risk for PCR-confirmed seasonal coronaviruses. We present transmission risk stratified by demographic features of households.

**Results: **We estimated that the proportion of cases acquired outside the home, weighted by age and region, was 90.7% (95% CI 84.6- 94.5, *n*=173/195) and within the home was 9.3% (5.5-15.4, 22/195). Following a symptomatic coronavirus index case, 14.9% (9.8 - 22.1, 20/134) of households experienced symptomatic transmission to at least one other household member. Onward transmission risk ranged from 11.90% (4.84-26.36, 5/42) to 19.44% (9.21-36.49, 7/36) by strain. The overall household secondary attack risk for symptomatic cases was 8.00% (5.31-11.88, 22/275), ranging across strains from 5.10 (2.11-11.84, 5/98) to 10.14 (4.82- 20.11, 7/69). Median clinical onset serial interval was 7 days (IQR= 6-9.5). Households including older adults, 3+ children, current smokers, contacts with chronic health conditions, and those in relatively deprived areas had the highest transmission risks. Child index cases and male index cases demonstrated the highest transmission risks.

**Conclusion: **Most seasonal coronaviruses appear to be acquired outside the household, with relatively modest risk of onward transmission within households. Transmission risk following an index case appears to vary by demographic household features, with potential overlap between those demonstrating the highest point estimates for seasonal coronavirus transmission risk and COVID-19 susceptibility and poor illness outcomes.

## Background

Understanding household transmission parameters is important for outbreak modelling and response as coronavirus disease 2019 (COVID-19) becomes established in communities worldwide. While studies are currently underway, developing robust estimates for household transmission parameters will take some time. Evidence from other human coronaviruses may therefore be useful, given similar routes of transmission ^1, 2^.

Outbreaks of other emerging coronaviruses, i.e. severe acute respiratory syndrome (SARS) and Middle East respiratory syndrome (MERS), appear to be primarily based in health care settings without substantial community transmission and household transmission studies are limited. Estimates of transmission risk to at least one other household member following a SARS index case range from 12.3%-13.5%, with estimated secondary household attack risks between 4.6%-10.2% ^3– 5^. In index cases, younger age and healthcare worker status were associated with lower risk of household transmission. A single study of MERS found that 44% of households with an index case experienced onwards transmission, with a secondary attack risk of 24% and risk factors for developing secondary infection in contacts including being adult, male, and having long-term conditions ^6^. 

Seasonal coronavirus infections also appear to cluster within households ^7^, with proportions of onwards transmission to at least one household member following child index cases ranging between 8% and 33% ^8, 9^. A recent community cohort study based in Michigan ^10^ found secondary attack risks ranging from 7.2%-12.6% for circulating seasonal coronavirus strains and clinical-onset serial intervals between 3.2-3.6 days.

Here we aimed to report key characteristics of seasonal coronavirus household transmission in a population-based UK cohort, using data from the Flu Watch study ^11^. We present the proportion of laboratory-confirmed seasonal coronavirus infections acquired within and outside the household, the household transmission risk and secondary attack risk for symptomatic cases, the clinical-onset serial interval, and transmission risk stratified by demographic household features.

## Method

### Study design and procedures

Data were drawn from the Flu Watch prospective cohort study of acute respiratory infections in English households ^11^. We included the three winter seasons (2006–2007, 2007–2008, 2008–2009), during which all samples were systematically tested for coronaviruses. The Flu Watch study methodology and cohort profile are described in detail elsewhere ^11, 12^.

Whole households were recruited annually to the study following random selection of a household member from GP practice lists. From 2008–2009, participants from the previous cohort were also re-invited to participate. Inclusion criteria were that all household members agreed to participate, and all members over 16 agreed to provide blood samples for other Flu Watch research. Exclusion criteria were household size >6 members, severely incapacitating or terminal illness in any household member, and heavy involvement in other research ^12^. 

Participants provided demographic data at seasonal baseline following recruitment, and were followed-up weekly via telephone or online throughout each season to report any symptoms of acute respiratory infection. If participants experienced any symptoms, they were requested to provide a nasal swab on the second day of illness and to provide a daily diary of symptoms from the first day of illness until the symptoms resolved. Real-time PCR was carried out to screen nasal swabs for a panel of viruses, including three circulating seasonal coronavirus strains (229-E, NL63, and OC43) ^11^.

### Definitions and analyses

Guided by an estimated incubation period of 2–5 days and further 2–18 days of symptomatic illness with viral shedding for seasonal coronaviruses ^13, 14^, we defined index cases as the first PCR-confirmed infection in a household or >23-days following a prior case, co-primary cases as potential index cases of the same strain arising within ≤2 days, and secondary cases as infections with the same strain occurring >2 days and ≤23 days from exposure to the index case(s). We assumed that household transmission to any secondary cases had occurred if the strain was unknown, but criteria for transmission were otherwise met. 

We estimated the proportions of total PCR-confirmed coronavirus cases in the study likely acquired in the community (i.e. index, co-primary, or single-person household cases) and acquired in the household (i.e. secondary cases). The proportions were weighted to the English national structure of age and region ^11^.

For households in which secondary transmission was possible (i.e. excluding single-person households and episodes of co-primary infection affecting all household members), we calculated the symptomatic household transmission risk– the proportion of households that experienced at least one secondary case – and the household secondary attack risk – the proportion of total exposed participants who became secondary cases. To avoid multiple inclusions of the same episode where there were co-primary cases, transmission risk and secondary attack risk were estimated based on a randomly-selected single index case. Due to the testing protocol, it was not possible to detect asymptomatic primary or secondary cases. We calculated the clinical-onset serial interval as the time in days from onset of reported symptoms in the index case(s) to onset of reported symptoms of the secondary case(s). All co-primary or multiple secondary cases in this cohort reported symptom onset on the same day.

We stratified the household symptomatic transmission risk by the following household features potentially relevant to transmission: the age structure of the household (adults between 16-64 only, older adult(s), adult(s) and children), age of index case (child <16, adult 16-64, older adult 65+) for households with children (not stratified for other household structures due to limited variation), the number of people and number of children in the household, whether the household contained current smoker(s), index of multiple deprivation, long-term health conditions in household contact(s), and the index sex, index healthcare worker status and index transmission-preventive hygiene behaviour. Index case hygiene behaviour was classified as a binary variable according to adherence to prevention guidelines ^15, 16^ i.e. covering the mouth while coughing or sneezing, using a single-use tissue, and washing the hands habitually after coughing or sneezing and at least moderately-frequently throughout the day (>=5 times according to median split and previous literature ^17^). Due to the small number of household transmissions, we present descriptive stratified analyses only and do not describe by strain sub-groups. Where features of the index case or index-exposed dyads were investigated, we included only those households where there was a clear index case rather than co-primary cases. Any instances of missing demographic data are noted in Table 1.

**Table 1.** Household transmission risk stratified by demographic features of household.

	Transmission Risk % (95% CI)	Transmission Occurred *n* (column %)	
		Yes	No
**Household structure**			
Adults only	9.09 (3.38, 22.21)	4 (20.00)	40 (35.09)
Older adult(s)	25.00 (10.47, 48.73)	5 (25.00)	15 (13.16)
Adult(s) and child(ren)	15.71 (8.84, 26.40)	11 (55.00)	59 (51.75)
**Number of children**			
0	14.06 (7.40, 25.10)	9 (45.00)	55 (48.25)
1	0.00 (n/a)	0 (0.00)	27 (23.68)
2	21.88 (10.60, 39.80)	7 (35.00)	25 (21.93)
3+	36.36 (13.47, 67.71)	4 (20.00)	7 (6.14)
**Smoker(s) in household** ^a^			
Yes	28.57 (10.61, 57.42)	4 (22.22)	10 (9.52)
No	12.84 (7.70, 20.66)	14 (77.78)	95 (90.48)
**IMD**			
1-2 (lower)	23.08 (10.50, 43.41)	6 (30.00)	20 (17.55)
3	12.20 (5.07, 26.53)	5 (25.00)	36 (31.58)
4	13.89 (5.78, 29.78)	5 (25.00)	31 (27.19)
5 (higher)	12.90 (4.80, 30.32)	4 (20.00)	27 (23.68)
**Contact(s) with chronic condition** ^b^			
Yes	16.67 (7.54, 32.92)	6 (33.33)	30 (27.03)
No	12.90 (7.41, 21.51)	12 (66.67)	81 (72.97)
**Index age** * **(households with children)**			
Adult	12.90 (4.75, 30.58)	4 (44.44)	27 (52.94)
Child	17.24 (7.11, 36.10)	5 (55.56)	24 (47.06)
**Index healthcare worker** *			
Yes	0.00 (n/a)	0 (0.00)	7 (6.60)
No	15.38 (9.85, 23.23)	18 (100.00)	99 (93.40)
**Index hygiene** ^* c^			
Yes (all recommendations)	0 (n/a)	0 (0.00)	13 (12.87)
No	16.98 (10.89, 25.50)	18 (100.00)	88 (87.13)
**Index sex** *			
Male	18.46 (10.69, 29.99)	12 (66.67)	53 (50.00)
Female	10.17 (4.57, 21.11)	6 (33.33)	53 (50.00)

* outbreaks with clear index case only ( *n*=10 outbreaks with co-primaries excluded); ^a^ unavailable for *n*=11 outbreaks; ^b^ unavailable for *n*=5 outbreaks; ^c^ unavailable for *n*=5 outbreaks; abbreviations: CI = confidence interval, IMD = indices of multiple deprivation.

To estimate potential non-detection of household transmissions due to non-adherence to swabbing protocol, we measured the proportion of exposed participants who reported symptoms within one month of an index case but did not submit a nasal swab. Analyses were performed in Stata Version 15.

## Results

### Proportion of infections acquired in the households and features of transmission

Table 2 reports the proportion of infections acquired within and outside of the household and the symptomatic transmission risk, secondary attack risk, and clinical-onset serial interval by strain and overall.

**Table 2.** Community and household-acquired infections and features of household transmission.

	229E	NL63	OC43	All *
**Infections acquired in community: % (95% CI, n/N)**	87.85 (73.40-94.99, 45/52)	93.82 (81.88-98.08, 55/60)	89.41 (77.91-95.29, 70/80)	90.66 (84.60-94.49, 173/195)
**Infections acquired in household: % (95% CI, n/N)**	12.15 (5.01-26.60, 7/52)	6.18 (1.92-18.12, 5/60)	10.59 (4.71-22.09, 10/80)	9.34 (5.51-15.40, 22/195)
**Household transmission risk: % (95% CI, n/N)**	19.44 (9.21-36.49, 7/36)	11.90 (4.84-26.36, 5/42)	13.21 (6.27-25.72, 7/53)	14.93 (9.78-22.11, 20/134)
**Secondary attack risk: % (95% CI, n/N)**	10.14 (4.82-20.11, 7/69)	5.10 (2.11-11.84, 5/98)	8.73 (4.56-16.10, 9/103)	8.00 (5.31-11.88, 22/275) **
**Serial interval (days): Mdn (IQR, range)**	7.0 (6.0-21.0, 5.0-21.0)	7.0 (6.0-7.5, 6.0-8.0)	6.5 (6-8.75, 6.0-14.0)	7.0 (6.0-9.5, 5.0-21.0)

*All strains include cases where strain was unknown (n=3); **due to unknown index strain, n=5 exposed contacts were included with no outbreak strain recorded.

There were a total of 195 coronavirus cases during the three seasons in households in which transmission was possible, of which a weighted proportion of 90.66% (95% CI 84.60-94.49, 173/195) were index or co-primary cases presumably acquired in the community, and 9.34% (5.51-15.40, 22/195) were in exposed household members and presumed to be acquired through household transmission. The proportion of presumed household-acquired infections ranged across strains from 6.18% (1.92-18.12, 5/60) for NL63, to 10.59% (4.71-22.09, 10/80) for OC43, and 12.15% (5.01-26.60, 7/52) for 229E. Strain data was unavailable for three cases.

There were 134 potential household outbreaks with a coronavirus index case or co-primary cases. All 22 co-primary cases from 10 households occurred on the same day. Of the 134 potential outbreaks, 22 had transmission to at least one other household member giving an overall household transmission risk of 14.93% (95% CI: 9.78-22.11). Across strains, household transmission risk ranged from 11.90% (4.84-26.36, 5/42) for NL63, 13.21% (6.27-25.72, 7/53) for OC43, and 19.44% (9.21-36.49, 7/36) for 229E.

A total of 22 exposed participants contracted a coronavirus infection, out of 275 participants at risk (excluding index and co-primary cases), yielding a household secondary attack risk of 8.00% (95% CI: 5.31-11.88, 22/275) overall. Secondary attack risks by strain were 5.10% (2.11-11.84, 5/98) for NL63, 8.73% (4.56-16.10, 9/103) for OC43, and 10.14% (4.82-20.11, 7/69) for 229E. Due to unknown index strain, outbreak strain was unavailable for five exposed participants.

The median clinical-onset serial interval was 7 days (IQR = 6-9.5 days, range 5-21 days). This median value was consistent for 229E and NL63, with some between-strain variation in range, and similar to the OC43 median serial interval of 6.5 days. There were two households for which the exact date of symptom onset of the exposed case could not be traced (only the beginning date of the week of illness), which were excluded from the serial interval calculation.

### Demographic features and transmission Risk

Table 1 reports household symptomatic transmission risk stratified by demographic features of households. Transmission risks were highest for households containing older adults (25.00%, 95% CI 10.47, 48.73), households with children (15.71%; 8.84, 26.40), then adult-only households (9.09%; 3.38, 22.21). Households with 3+ children (36.36%; 13.34, 67.96), those with smokers (28.57%; 10.61, 57.42), those in deprived areas (23.08%; 10.50, 43.41), and those in which 1+ household contact had a chronic health condition (16.67%; 7.54, 21.51) had the highest transmission risks within their categories. In households with children, child index cases demonstrated higher transmission risk (17.24%; 7.11, 36.10) than adult index cases (12.90%; 4.75, 30.58). Male index cases also demonstrated higher transmission risk (18.46; 10.69, 29.99) than female index cases (10.17%; 4.57, 21.11). There was no evidence of onward transmission if the index case was a healthcare worker or if they practiced good hand and respiratory hygiene, though the number of index cases in these categories were low ( *n*= 7 and 13 respectively).

### Adherence to swabbing protocol during household outbreaks

Among the 275 exposed household members, there were 75 distinct episodes of respiratory symptoms within 23 days of a household coronavirus index case. For 4 of these episodes (5.33%), the participant did not provide a swab at any point during their illness.

## Discussion

The study describes a range of important characteristics of seasonal coronavirus transmission that have not previously been reported. The great majority of infections (91%) were acquired outside the household. Following a symptomatic index case within the household, onward transmission to at least one other household member occurred in 14.9% of households, with a secondary attack risk of 8.0%. Risk of onwards household transmission fell within previous estimates for seasonal coronaviruses ^8, 9^ and were higher than the limited literature suggests for SARS ^3– 5^, but lower than for MERS ^6^. The secondary attack risk fell within the range identified for seasonal coronavirus strains in a recent US-based longitudinal cohort study ^10^ and was somewhat lower than preliminary estimates of the household secondary attack risk for COVID-19 - ranging between 10.5% in USA ^18^, 13.6% in Taiwan ^19^; 14.9% in Shenzen, China ^20^, and 31.6% in Zhejiang Province, China ^21^. Our estimate for the median clinical-onset serial interval (7 days) was larger than Monto *et al.*’s (2020) estimate ^10^ (3.2-3.6 days), though this may reflect different handling of potentially co-primary cases, as only an upper-bound distance between cases was specified in the latter study. 

Since the virus causing COVID-19 is a recently emerged pathogen there is likely to be minimal population immunity, this contrasts with seasonal coronaviruses, where results from the Flu Watch study show a protective effect of recent infection ^22^. This may contribute to higher household secondary attack risk in COVID-19 than in seasonal coronavirus. Given emerging evidence that some individuals with asymptomatic or pre-symptomatic SARS-CoV-2 infections may be capable of transmitting the virus to others ^23– 26^, preventing household transmission of SARS-CoV-2 may be notably challenging. Recent COVID-19 household transmission studies have also occurred during periods when there have been stringent measures to control transmission. Intensified hand and respiratory and environmental cleaning during a pandemic may decrease household transmission. Measures that ensure families stay at home together may increase household secondary attack risk by increasing the level of contact in the home. In the current study the majority of infections were acquired outside the household. The proportion of infections acquired outside the household is likely to be lower during periods of social distancing ^27^. We have previously shown that conducting a wide range of activities outside the household, including visiting supermarkets, shops, restaurants, places of worship and using public transport, increase the risk of acquiring acute respiratory infection ^28^.

Demographic and health-related characteristics with the highest point estimates for symptomatic transmission risk overlapped with risk groups for susceptibility and/or poor outcomes in COVID-19, namely households comprising older adults, current smokers, exposed members with chronic illness, and those in deprived areas ^29– 31^. Although children had a higher point estimate for transmission risk, index cases were distributed relatively evenly across age groups, in contrast with a Michigan community cohort study in which children comprised the majority of index cases for most strains ^10^. Good hand and respiratory hygiene by index cases may be helpful for disrupting transmission, possibly overlapping with the lack onwards transmission from healthcare workers. The findings should be interpreted with caution given relatively small numbers of positive transmissions and, related, wide confidence intervals for transmission risk estimates. We were underpowered to conduct multivariate analysis required to disentangle the interrelationships between potential demographic and health-related risk factors and this is a relevant area for further research concerning seasonal and pandemic coronaviruses.

This study had a number of limitations. Data were limited to winter seasons. Only symptomatic cases were swabbed and detected. While there appeared to be good adherence to the swabbing protocol among household members exposed to an index case, five percent of those who developed symptoms following exposure failing to provide a swab so secondary transmission may be underestimated. It is possible that the nasal swabbing protocol, which was developed primarily for influenza detection, did not provide ideal sensitivity for these strains of coronavirus. We also cannot exclude that index cases in the initial weeks of the study could have acquired the case through undetected household transmission. The analyses did not account for background rate of infection in the community and consequently some apparent secondary cases may have been acquired incidentally outside of the household. All risk factor analyses were limited by a relatively small number of onward transmissions.

To our knowledge, this study presents the first investigation into household transmission in seasonal coronaviruses in a population-based UK sample. In typical winter seasons with individuals freely able to continue typical activities and social contacts, household transmission appears to be lower than in COVID-19. However, this difference may reflect different levels of population immunity and behaviours rather than intrinsic differences in the transmissibility of seasonal coronaviruses and the pandemic strain. The high proportion of infections acquired outside the household reinforces Stay at Home messages. Corresponding population-based studies investigating COVID-19 household transmission - such as the UK Virus Watch study, modelled on the Flu Watch study used here - are warranted to inform transmission models and public health interventions. 

### Ethics statement

The protocol was approved by the Oxford Multi-Centre Research Ethics Committee (06/Q1604/103).

## Data availability

### Underlying data

University College London: Household Transmission of Seasonal Coronavirus Infections: Results from the Flu Watch cohort study. https://doi.org/10.5522/04/12383873.v1

This project contains the following underlying data:

- Household_CoV_acquired.csv (data required to compute the proportion of cases presumably acquired outside of the household versus and the proportion acquired from household transmission. Each row represents an anonymised PCR-confirmed seasonal coronavirus case)
- Household_CoV_acquired.dta (above data file in .dta format)
- Household_CoV_TransmissionRisk.csv (data required to compute the risk of symptomatic onward household transmission following a seasonal coronavirus index case, and perform stratified descriptive analyses)
- Household_CoV_TransmissionRisk.csv (above data file in .dta format)
- Household_CoV_SAR.csv (data required to compute the seasonal coronavirus secondary attack risk overall and by strain. Each row represents an anonymised exposed-index pair from a given outbreak)
- Household_CoV_SAR.dta (above data file in .dta format)
- HH Transmission Serial Interval.csv (presents available, anonymised data required to compute the median clinical-onset serial interval overall and by strain for each household outbreak)

Data are available under the terms of the Creative Commons Attribution 4.0 International license (CC-BY 4.0).
