# Supplementary material for: Household transmission of seasonal coronavirus infections: Results from the Flu Watch cohort study
Source: Wellcome Open Res. 2020 Jun 19;5:145. [Version 1] doi: 10.12688/wellcomeopenres.16055.1 (PMC7848853; doi:10.12688/wellcomeopenres.16055.1)
Supplement: Peer review history

# Peer review: Reviewer response for version 1

**DOI:** 10.21956/wellcomeopenres.17611.r41693

**Published:** 2021-01-29

**Authors:** Hannah Kirking, Melisa Shah

**Affiliations:** 1 COVID-19 Response Team, Centers for Disease Control and Prevention, Atlanta, GA, USA

**Brief Summary:**

 This is a well-written and timely article on household transmission of seasonal coronaviruses with implications for the current pandemic. This presents original work using a population-based cohort in England which has previously been described. This prospective cohort was contacted weekly during the winter season, which is when seasonal coronaviruses circulate. Symptomatic participants in each household kept a daily log of symptoms allowing for granular data collection. PCR and strain level characterization of coronavirus diagnoses were included which was a major strength. Household transmission risk is presented and stratified by relevant characteristics including household structure, number of children, smoking, and hygiene. The findings include description of clinical serial interval, secondary attack risk, and household transmission risk stratified by strain type. The comparison of this study’s household attack rates to other coronaviruses (including SARS and MERS) is very valuable and adds to the cogwheel of data informing household transmission of COVID-19. The authors do a good job of noting caution in interpretation of results given that the risk factor section is underpowered with overlapping confidence intervals.

**Major Concerns:**

 A particularly interesting result is the increased risk of transmission among males, households with older adults, smokers, and chronic illnesses which is also seen as risk factors for severe disease in COVID-19. But looking at Table 1, it is difficult to know if these trends are statistically significant given the overlapping confidence intervals. Additionally, for “older adults in the household” it is unclear whether this may simply be a proxy for more individuals in a household or for having children in the household (such as a multigenerational family). The limitation of wide confidence intervals is raised in the discussion section, but this potential relationship also appears in the abstract conclusion as a major finding. Given that there are so many caveats and need for further work in this area, I suggest removing the finding from the abstract and keep it described with all the caveats in the discussion section. The fact that there were no household transmission when the index case followed hand and respiratory hygiene protocols seems to be a really important data point and finding of the study to highlight.

**Minor Concerns: **

1. Please define “deprived areas” or IMD (indices of multiple deprivation) used in Table 1 and comments in the discussion (bottom left paragraph) in the methods section.
2. In the methods section, the inability to identify asymptomatic primary or secondary cases is raised. It would be helpful to include this limitation in the discussion section and provide any underlying literature on asymptomatic transmission of seasonal coronaviruses. This is an important piece in how such data is linked to transmission of the current pandemic strain.
3. One of the strengths of the paper is the description of strains, but the discussion does not include any background on known differences or comments/hypotheses regarding whether strain type may play a role in household transmission risk.
4. When discussing “households with older adults”, does this also include children and could this simply be a proxy for more people in the household?
5. It may be valuable to discuss possible background immunity to common hCoVs a bit more in the discussion, and its possible impact on household transmission findings. This is where there is also a big difference between the common hCoVs and SARS-CoV-2. Authors allude to this throughout, but it may benefit from some attention in the discussion section.

Is the work clearly and accurately presented and does it cite the current literature?

Yes

If applicable, is the statistical analysis and its interpretation appropriate?

Partly

Are all the source data underlying the results available to ensure full reproducibility?

Yes

Is the study design appropriate and is the work technically sound?

Yes

Are the conclusions drawn adequately supported by the results?

Yes

Are sufficient details of methods and analysis provided to allow replication by others?

Yes

Reviewer Expertise:

Respiratory virus transmission, SARS-CoV-2, household transmission investigations.

We confirm that we have read this submission and believe that we have an appropriate level of expertise to confirm that it is of an acceptable scientific standard.

# Peer review: Reviewer response for version 1

**DOI:** 10.21956/wellcomeopenres.17611.r41764

**Published:** 2021-01-25

**Authors:** Melissa Rolfes

**Affiliations:** 1 Influenza Division, Centers for Disease Control and Prevention, Atlanta, GA, USA

The authors provide a report on seasonal coronavirus infections detected in the Flu Watch cohort in the UK and examine household and demographic features associated with subsequent infection in other household members. In general, the rationale and implications of the study are clearly described and further elaboration of the methods and results would strengthen the manuscript overall. This is important work for the public health community, grappling with the ongoing SARS-CoV-2 pandemic; however, one limitation of the Flu Watch study, and its conclusions and relevance for the current pandemic, are that all detected infections were symptomatic because only symptomatic participants contributed nasal swab specimens for PCR testing. I look forward to further studies that can build on this study design to innovatively capture asymptomatic seasonal respiratory virus infections to better understand the drivers behind onward transmission.

 Specific comments that I hope the authors can address in the next version are below.

 Major comments

- Please include further description of the weekly telephone/internet survey, including the list of symptoms that were asked about. The description should include enough detail to answer the following questions: Was the question addressed to one member of the household or did each member have to respond about symptoms? Was the list of symptoms different if the participant was a young child (and below what age were these different symptoms solicited)?
- The methods section did not include a definition of the indices of multiple deprivation. A short description of what the indices are based on would be important for re-creating this work and analysis. Additionally, a reference to the index would be helpful for readers.
- Please include the method you used to estimate the confidence intervals for the household transmission risk, the symptomatic secondary transmission risk, and the community infection risk…well, all really.
- The results section could be elaborated with more information about the cohort size. How many people (and how many households) participated in each season? What were the demographic, household, geographic, and socioeconomic characteristics of the participants?
- In the 3 ^rd^ paragraph of the results section, the authors state that 22 households had transmission (out of a possible 134) = 16.42% household transmission risk. But in Table 2, the number is 20 out of a possible 134 = 14.93%. Please make the appropriate corrections in the table and text.
- The literature on household transmission of SARS-CoV-2 is moving quickly, but it’d be useful to update the Discussion section and references to include more recent estimates of household secondary transmission risk for SARS-CoV-2.

Minor comments

- The authors appropriately use the term “symptomatic transmission risk”, but perhaps the authors could consider modifying their title to further remind readers. Something like “Household transmission of symptomatic coronavirus infections: …”
- To avoid including both co-primary cases in the analysis, the authors chose a randomly-selected case to serve as the “primary”/index case. Can you include in the discussion whether or not your findings (particularly the risk %s in Table 1) were sensitive to this random draw? Effectively what you’re doing is imputing who the primary case was in the household, but you’ve done the imputation once in the current analysis. You could do the imputation multiple times (10-15 times, for example) and then summarize the transmission risks that you get in each of those iterations (just like you would in a multiple imputation). This approach might be more robust to chance findings, especially since there were quite a few co-primary cases.
- I appreciate the importance of weighting the infection proportions for your sampling scheme and to the underlying population. In Table 1 and 2, though, it would have been helpful to have a reminder of which proportions were weighted to the underlying population and which ones were not. I believe you’d be able to weight the first two rows, but not the others; however, you’d know best. A footnote would suffice to me.

Is the work clearly and accurately presented and does it cite the current literature?

Yes

If applicable, is the statistical analysis and its interpretation appropriate?

Yes

Are all the source data underlying the results available to ensure full reproducibility?

Yes

Is the study design appropriate and is the work technically sound?

Yes

Are the conclusions drawn adequately supported by the results?

Yes

Are sufficient details of methods and analysis provided to allow replication by others?

Partly

Reviewer Expertise:

Epidemiologic methods, epidemiology of influenza and other respiratory viruses, household transmission.

I confirm that I have read this submission and believe that I have an appropriate level of expertise to confirm that it is of an acceptable scientific standard, however I have significant reservations, as outlined above.

# Peer review: Reviewer response for version 1

**DOI:** 10.21956/wellcomeopenres.17611.r40663

**Published:** 2020-10-29

**Authors:** Eben Kenah

**Affiliations:** 1 Department of Biostatistics, School of Public Health, Ohio State University, Columbus, USA

This is a fascinating study of the household transmission of seasonal coronavirus infections in a well-described and well-run cohort study. The associations between transmission risk and the age of household members, number of children, male primary cases, primary case hygiene, smoking, and deprivation are all plausible and may be useful for thinking about the transmission of COVID-19. There are far too few studies of this kind, so this one is useful both for its own results and as an example for others. The paper is clearly written, and the data analyses are simple but thoughtful. My major comments are

- The methods used to calculate confidence intervals should be described more clearly, and confidence intervals (e.g., exact confidence intervals) should be given even when no onward transmission was observed.
- The limitations are adequately discussed with one exception: the methods implicitly assume that all secondary cases are infected by the primary case. Because it neglects multiple generations of transmission within the household.

Additional minor comments are listed below:

1. I appreciate the consistent use of "secondary attack risk''.
2. In line with the usage recommended by Giesecke (2014) ^1^, it might be better to use "primary case'' instead of "index case'' for the first person infected in the household. This would also be more consistent with the use of "co-primary'' throughout the manuscript.
3. (page~4, Table~1) Exact confidence intervals can be given for household groups in which there were no observed transmissions.
4. (page~5, bottom or right column) It would be more accurate to say that no observed onward transmission if the primary case was a healthcare worker or if they practiced good hand and respiratory hygiene.
5. The methods used to calculate confidence intervals need to be explained more clearly. When I try to replicate them, I get similar results but not exact matches.
6. Given the uncertainty in the point and interval estimates, a single decimal point in the percentages is probably sufficient.
7. One other limitation needs to be mentioned: The method of calculating the secondary attack risk implicitly assumes that all secondary cases are infected by the primary case, ignoring the possibility of multiple generations of transmission within the household.

Is the work clearly and accurately presented and does it cite the current literature?

Yes

If applicable, is the statistical analysis and its interpretation appropriate?

Yes

Are all the source data underlying the results available to ensure full reproducibility?

Yes

Is the study design appropriate and is the work technically sound?

Yes

Are the conclusions drawn adequately supported by the results?

Yes

Are sufficient details of methods and analysis provided to allow replication by others?

Partly

Reviewer Expertise:

Statistical methods for infectious disease epidemiology, epidemiologic methods, survival analysis, causal inference.

I confirm that I have read this submission and believe that I have an appropriate level of expertise to confirm that it is of an acceptable scientific standard, however I have significant reservations, as outlined above.

## References

[1] : Primary and index cases. *The Lancet*.2014;384(9959) : 10.1016/S0140-6736(14)62331-X 10.1016/S0140-6736(14)62331-X 25483164
